# Tracheal Extubation Under Deep Anesthesia Using Transnasal Humidified Rapid Insufflation Ventilatory Exchange vs. Awake Extubation: An Open-Labeled Randomized Controlled Trial

**DOI:** 10.3389/fmed.2022.810366

**Published:** 2022-03-03

**Authors:** Jin Qiu, Mian Xie, Jie Chen, Bing Chen, Yuanjing Chen, Xiwen Zhu, Hui Lin, Tao Zhu, Guangyou Duan, He Huang

**Affiliations:** ^1^Department of Anesthesiology, The Second Affiliated Hospital, Chongqing Medical University, Chongqing, China; ^2^Chongqing Traditional Chinese Medicine Hospital, Chongqing, China; ^3^Department of Statistics, Army Medical University, Chongqing, China; ^4^Department of Anesthesiology and Translational Neuroscience Center, West China Hospital, Sichuan University, Chengdu, China

**Keywords:** tracheal extubation, hypoxia, THRIVE, deep anesthesia, awake extubation

## Abstract

**Background:**

Tracheal extubation can be associated with several complications, including desaturation, agitation, hypertension, and tachycardia. We hypothesize that the use of transnasal humidified rapid insufflation ventilator exchange (THRIVE) immediately after extubation under deep anesthesia reduces the incidence of these adverse events.

**Methods:**

One hundred patients who underwent elective abdominal surgery under general anesthesia were randomly assigned to undergo tracheal extubation under deep anesthesia employing THRIVE (THRIVE group) or awake extubation (CONTROL group). The primary outcome was the incidence of experiencing desaturation (SpO_2_ < 90%) at any time during emergence from anesthesia. Secondary outcomes included variations in heart rate and blood pressure, comfort level, bucking, and agitation.

**Results:**

The THRIVE group showed a lower incidence of desaturation than the CONTROL group (12 vs. 54%, OR = 0.22 [95% CI, 0.10–0.49], *P* < 0.001). Less patients in the THRIVE group experienced a 20% (or more) increase in mean arterial pressure (4 vs. 26%, OR = 0.15 [95% CI, 0.04–0.65], *P* = 0.002). THRIVE patients did not suffer from agitation or bucking, while in the CONTROL group agitation and bucking occurred in 22 and 58% of the patients, respectively. Additionally, the THRIVE group showed a lower incidence of uncomfortable experience than the CONTROL group (8 vs. 36%, OR = 0.22 [95% CI, 0.08–0.61], *P* = 0.001).

**Conclusion:**

Tracheal extubation under deep anesthesia using THRIVE decreases the incidence of desaturation and adverse haemodynamic events and increases patient satisfaction. Extubation under deep anesthesia using THRIVE might be an alternative strategy in selected patient populations.

## Introduction

Following surgery under general anesthesia, tracheal extubation is often performed when the patient is fully awake with full recovery of spontaneous breathing (1). Emergence from anesthesia following tracheal extubation can be associated with desaturation, coughing, agitation, hypertension, and tachycardia, all of which decrease the patients' quality of recovery (2–5). Furthermore, one study has demonstrated that the majority (67%) of severe complications related anesthesia such as myocardial infarction began at the end of surgery and emergence from anesthesia (6). Thus for those with severe cardiovascular or respiratory disease or neurosurgical patients, smooth emergence from anesthesia is highly desired, and various strategies of reducing the response to the tracheal tube during emergence from general anesthesia are explored (7, 8). Among these methods, extubation under deep anesthesia but with maintained spontaneous breathing was advocated to prevent the unwanted side effects of awake tracheal extubation, contributing to the aforementioned adverse events (9–11). Previous clinical studies have shown that extubation under deep anesthesia decreases the stress response and improves the level of comfort (12–14). However, tracheal extubation in the unconscious patients increases the risk of hypoxaemia and prolongs recovery time (15, 16).

Transnasal humidified rapid insufflation ventilator exchange (THRIVE) is a new apnoeic oxygenation technique combining high-flow heating and humidified oxygen therapy to prolong apnoea time (17–19). A previous study demonstrated that THRIVE allows for a median apnoea time of 20–45 min (20, 21). And as reported in several studies, THRIVE secured adequate oxygenation during induction and maintenance of anesthesia employing target-controlled propofol infusion and deep neuromuscular blockade in patients undergoing laryngo-microsurgery (22–24). We hypothesize, that tracheal extubation under deep anesthesia using THRIVE can effectively decrease the risk of hypoxaemia, agitation, hypertension and tachycardia, providing a more comfortable experience to patients following emergence from general anesthesia compared to awake extubation. Thus, this study aimed to include a selected patient population to determine the safety and effect of such extubation strategy during emergence from general anesthesia.

## Methods

### Study Design

This open-labeled, randomized, controlled, and parallel (allocation ratio 1:1) study was performed at the Second Affiliated Hospital, Chongqing Medical University from October 23, 2020, to December 24, 2020 following the CONSORT 2010 guidelines. The study protocol was registered in the Chinese Clinical Trial Registry (www.chictr.org.cn, registration number: ChiCTR2000040050) and was approved by the Hospital Ethics Committee of The Second Affiliated Hospital, Chongqing Medical University (Approved ID: 2020-110). All patients provided written informed consent.

### Patients

Patients aged 18–65 years with an American Society of Anesthesiology (ASA) physical status I–III scheduled to receive elective abdominal surgery were included. Exclusion criteria were body mass index > 30 kg/m^2^, known or estimated difficult airway, history of severe chronic obstructive pulmonary disease (severity grade III or IV), severe or uncontrolled bronchial asthma, pulmonary infection, bronchiectasis, thoracic deformity and intrathoracic diseases (e.g., mediastinal and thoracic tumors), acute coronary syndrome, persistent ventricular tachyarrhythmia (NYHA grade III or IV), severe heart disease, cirrhosis and ascites with Child Pugh grade B or C, chronic renal failure stage 4 or 5 requiring dialysis, severe neuromuscular diseases, having undergone airway surgery or surgery around the airway (including thyroid surgery), with high risk of aspiration and inability to communicate.

### Randomization and Masking

Computer-generated randomization sequence employing opaque sealed envelopes with a 1:1 allocation was done by an independent epidemiologists. All participants, anaesthesiologists and surgeons were blinded to group allocation during treatment in the operating room (OR). Data collection was performed by trained professionals not involved in patient care and an independent statistician blinded to group allocation performed the data analysis.

### Anesthesia and Analgesia

For all included patients, invasive blood pressure, pulse oximetry (SpO_2_), non-invasive cardiac output (CNAP Monitor 500, CN Systems Medizintechnik GmbH, Austria), and end-expiratory carbon dioxide oxygen were continuously monitored after entering the OR. Anesthesia depth was assessed based on the patient state index (PSI) using Masimo Radical 7 (Masimo Corp., Irvine, CA, USA). Anesthesia was induced with intravenous midazolam (0.05–0.2 mg/kg), sufentanil (0.5–2 μg/kg), propofol (1.0–2.0 mg/kg), and rocuronium (0.6–1.2 mg/kg). The patients trachea was intubated when PSI dropped to 40–50. Maintenance of a combined intravenous-inhalation anesthesia was done with sevoflurane (1–3%), remifentanil (0.2–0.4 μg/kg/min), and propofol (4–12 mg/kg/h), yielding at a PSI of 25–50 and a mean arterial blood pressure (MAP) and heart rate within ±20% of baseline. Intraoperatively, patients received intravenous boluses of 0.2 mg/kg rocuronium as required. Postoperative pain control was immediately started after surgery employing a patient-controlled analgesia (PCA) pump with sufentanil (2.5 μg/ml). PCA consisted of a loading dose of 2 ml, background infusion of 1.5 ml/h, bolus dose of 1 ml, lockout period of 10 min, and maximal dose of 12 ml/h.

### Study Intervention

Following admission to the PACU, muscle relaxation was assessed by train-of-four (TOF) stimulation using quantitative neuromuscular monitoring (IntelliVue MX700, Philips, Germany). Pulmonary volume was monitored using Electrical Impedance Tomography (EIT, PulmoVista 500, Draeger, Germany). Additionally, PSI, rSO2L (left regional cerebral oxygen saturation), and rSO2R (right regional cerebral oxygen saturation) were continuously monitored in the PACU.

For patients in the THRIVE group, a standardized management for tracheal extubation was followed. (1) Secretions from the trachea and oral cavity were immediately sucked at admittance to the PACU; (2) Tracheal extubation was performed in the unconscious patients employing THRIVE (60 L/min, FiO_2_ 60%, 36°C, Mindray SV300, Mindray, China); (3) THRIVE was maintained until the patient was able to follow instructions with a PSI of at least 75, a TOF of more than 90% and adequate spontaneous breathing. After THRIVE termination, patients breathed room air under closely monitoring. In case of SpO_2_ < 90%, first jaw thrust was applied for 3 min. When jaw thrust did not lead to an increase of oxygenation with a SpO_2_ above 90% a cuffed oropharyngeal airway was installed followed by re-intubation when deemed necessary.

For the CONTROL group, tracheal extubation was performed in the conscious patient able to follow instructions with a PSI of at least 75, a TOF of more than 90% and adequate spontaneous breathing. And after extubation, patients breathed room air as same as the THRIVE group under closely monitoring. When SpO_2_ decreased below 90% after tracheal extubation, the patient was first asked to take a deep breath. If this did not increase SpO_2_, oxygen supplementation via a nasal cannula followed by jaw thrust, installation of a cuffed oropharyngeal airway, and ultimately re-intubation was considered. All patients stayed in the PACU until Steward Score reached 4.

### Outcomes

SpO_2_, heart rate, and MAP were continuously monitored in the OR and in the PACU. The primary outcome was the incidence of desaturation in the PACU, which was defined as SpO_2_ < 90% at any time as reported in previous studies (25–27). Secondary outcomes included bucking, agitation, hypertension (an increase of ≥20% in MAP from the baseline) and tachycardia (an increase of ≥20% in heart rate from the baseline). Heart rate and MAP were recorded before and 30 s after tracheal extubation. Scores of bucking [0: No coughing; 1: Minimal coughing (once or twice); 2: Moderate coughing (3-4 times); 3: Severe coughing (≥5 times)] (5) during the patients' stay in the PACU was evaluated, and bucking was recorded as score ≥ 1. Four-point agitation score (1: calm; 2: not calm but could be easily calmed; 3: not easily calmed, moderately agitated, or restless; and 4, combative, excited, or disoriented) (28) was also recorded during the patients' stay in the PACU. Other adverse events in PACU are also recorded, such as aspiration, airway injury, bleeding, reintubation, etc. At discharge from the PACU, patients were asked to rate their stay in the PACU as “good,” “average,” or “bad.”

Arterial blood gas analysis was performed before surgery, when entering the PACU, 10 min after tracheal extubation, and at discharge from the PACU. PaO_2_, PaCO_2_, PH, SaO_2_, HCO3^−^, and Lac were determined. Respiratory rate, region of interests1(ROI1), region of interests2(ROI2), region of interests3(ROI3), region of interests4(ROI4), changes in end-expiratory lung impedance (ΔEELI1, ΔEELI2, ΔEELI3, ΔEELI4, and ΔEELIGlobal) before surgery, when entering the PACU, 10 min after tracheal extubation, and at discharge from the PACU were recorded based on EIT. Further, Qor-15 was used to evaluate the patients' postoperative recovery. Postoperative pulmonary complications, laryngalgia (yes/no), hoarseness (yes/no), and the duration PACU stay and length of hospital stay were recorded.

### Statistical Analysis

#### Sample Size Calculation

Based on our own pilot observation in the PACU, the incidence of desaturation (SpO_2_ < 90%) was 55% for awake extubation (CONTROL group), which is similar with existing literature (29, 30). We hypothesized that THRIVE reduces the incidence of desaturation following tracheal extubation by 30%. Based on a significance level of 0.05 and a power of 0.8, 40 patients in each group were required. Considering a missing follow-up rate of ~20%, the total required sample size was 100 patients, with 50 patients in each group. Sample size was calculated based on a 1:1 parallel control study using the sample size calculation software PASS version 11.0 (NCSS, Kayesville, UT).

Data were presented as absolute numbers (percentage), mean (standard deviation), or medians (interquartile range). Analysis was performed based on the intention-to-treat principle. Between-group comparisons of discontinuous variables were done using the chi-squared test or Fisher's exact test. In addition, agitation, bucking, and uncomfortable experience was expressed as odds ratio (OR) with 95% confidence intervals (CI). Between-group comparison of normally distributed continuous variables were done using the independent-samples *T*-test. Moreover, between-group differences of non-normal distributed data were assessed using the Mann-Whitney *U*-test. For continuous variables at different time points, a two-way repeated measures analysis of variance (ANOVA) was performed. Statistical analyses were performed using SPSS software 23.0 (SPSS, Chicago, IL, USA). Statistical significance was set at a two-sided *P* < 0.05.

## Results

**Figure 1:**
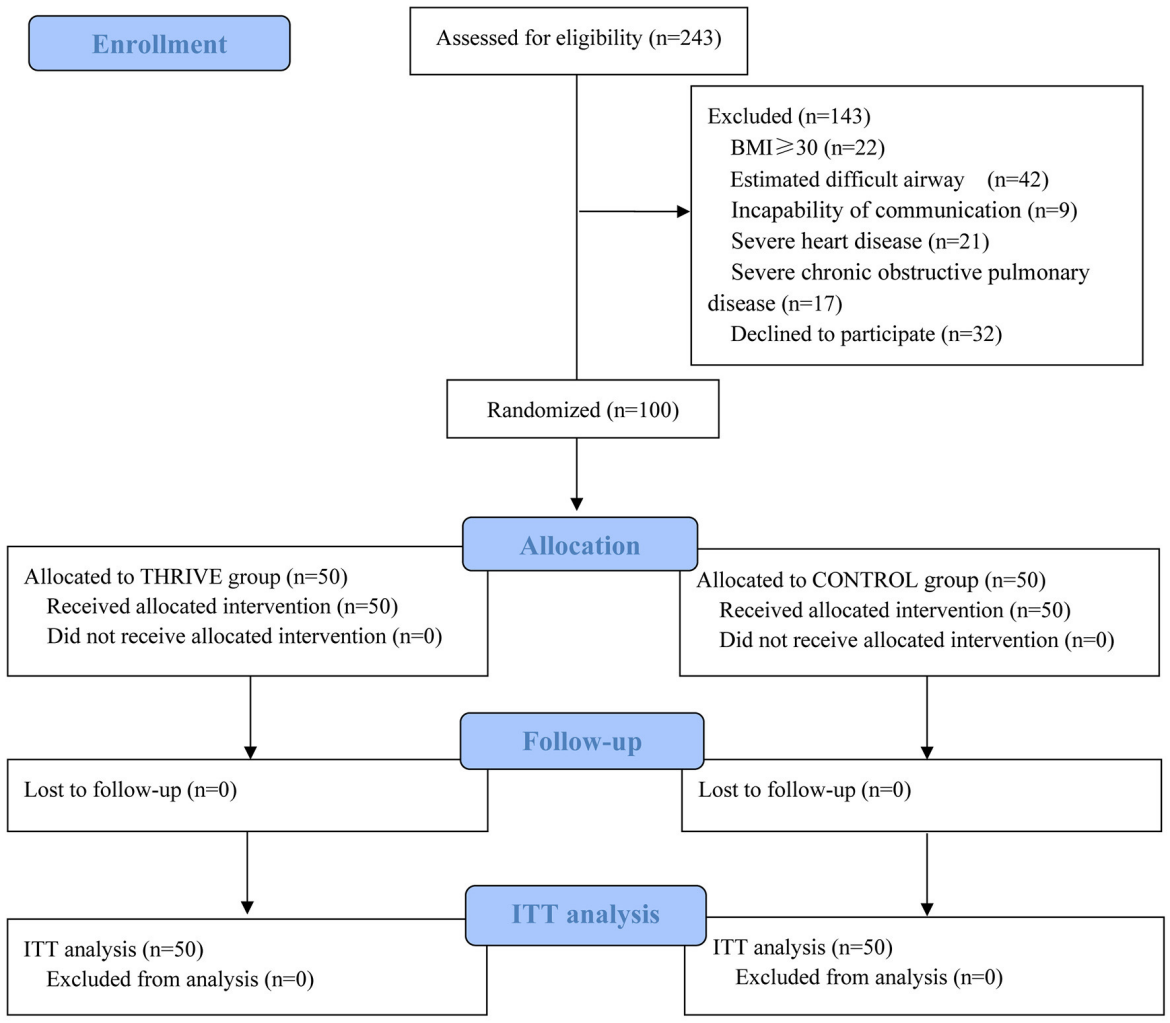
Flow diagram of the study. ITT, intention to treat analysis.

As shown in Figure 1, 243 patients were screened for eligibility. Based on the inclusion and exclusion criteria, 100 patients were randomly allocated to the THRIVE and CONTROL groups (*n* = 50, in each group). No patients were lost to follow-up. All included patients completed the study conform study protocol; Accordingly 100 patients were included in the final analysis. Except for basal heart rate, there was no between-group difference in the baseline or intraoperative characteristics (Table 1). The time between PACU admission and extubation in the CONTROL group was 23.1 ± 13.8 min, and the time between PACU admission and THRIVE termination in the TRIVE group was 23.0 ± 7.4 min.

**Table 1:** Baseline demographic characteristics, preoperative and intraoperative data between patients in THRIVE and CONTROL groups.

	**THRIVE group (*n* = 50)**	**CONTROL group (*n* = 50)**	***P***
Age (year)	45.0 ± 11.5	47.3 ± 11.5	0.319
Sex (male/female)	17/33 (34/66%)	16/34 (32/68%)	0.832
Height (cm)	161.4 ± 6.6	161.1 ± 8.3	0.811
Weight (kg)	61.1 ± 8.9	62.1 ± 12.3	0.649
Body max index (kg/m^2^)	23.4 ± 2.6	23.8 ± 3.6	0.512
Surgical method (endoscope/open)	41/9	41/9	1.000
American Society of Anesthesiologists class (I/II/III)	2/42/6 (4/84/12%)	0/43/7 (0/86/14%)	0.352
Mallampati class (I/II)	11/39 (22/78%)	15/35 (30/70%)	0.362
Cormack-Lenhane class (I/II)	47/3 (94/6%)	45/5 (90/10%)	0.461
Surgery duration (min)	105.6 ± 76.2	120.6 ± 72.3	0.317
Blood loss (ml)	106.1 ± 176.9	104.4 ± 164.6	0.960
Mean arterial pressure (mmHg)	98.4 ± 12.2	97.4 ± 12.5	0.694
Heart rate (bpm)	74.1 ± 10.5	79.2 ± 11.7	0.024
Cardiac output (L/min)	5.4 ± 0.9	5.8 ± 1.4	0.078
Systemic vascular resistance (dyn.s/cm^5^)	1326.0 ± 311.8	1259.6 ± 282.1	0.273
Stroke volume (ml)	72.9 ± 11.3	72.5 ± 11.2	0.854
Stroke volume variation (%)	9.6 ± 4.2	9.3 ± 5.1	0.720
Patient state index	94.6 ± 2.3	94.6 ± 2.6	0.935
rSO_2_L (%)	84.2 ± 2.6	86.1 ± 3.9	0.113
rSO_2_R (%)	83.3 ± 1.9	83.9 ± 2.9	0.526
Respiratory rate (bpm)	15.9 ± 4.1	14.8 ± 3.5	0.139
PaO_2_ (mmHg)	140.2 ± 87.8	126.7 ± 73.2	0.409
PaCO_2_ (mmHg)	38.1 ± 3.6	36.9 ± 3.7	0.133
SaO_2_ (%)	99.1 ± 0.8	99.0 ± 0.9	0.729
HCO3- (mmol/L)	24.4 ± 2.4	23.7 ± 2.2	0.126
Lac (mmol/L)	0.93 ± 0.34	0.94 ± 0.43	0.918
SPO_2_ (%)	98.8 ± 1.9	98.9 ± 1.2	0.800

*ASA, American Society of Anesthesiologists; SVR, Systemic vascular resistance; Data were presented as mean (SD) or number (proportion)*.

**Figure 2:**
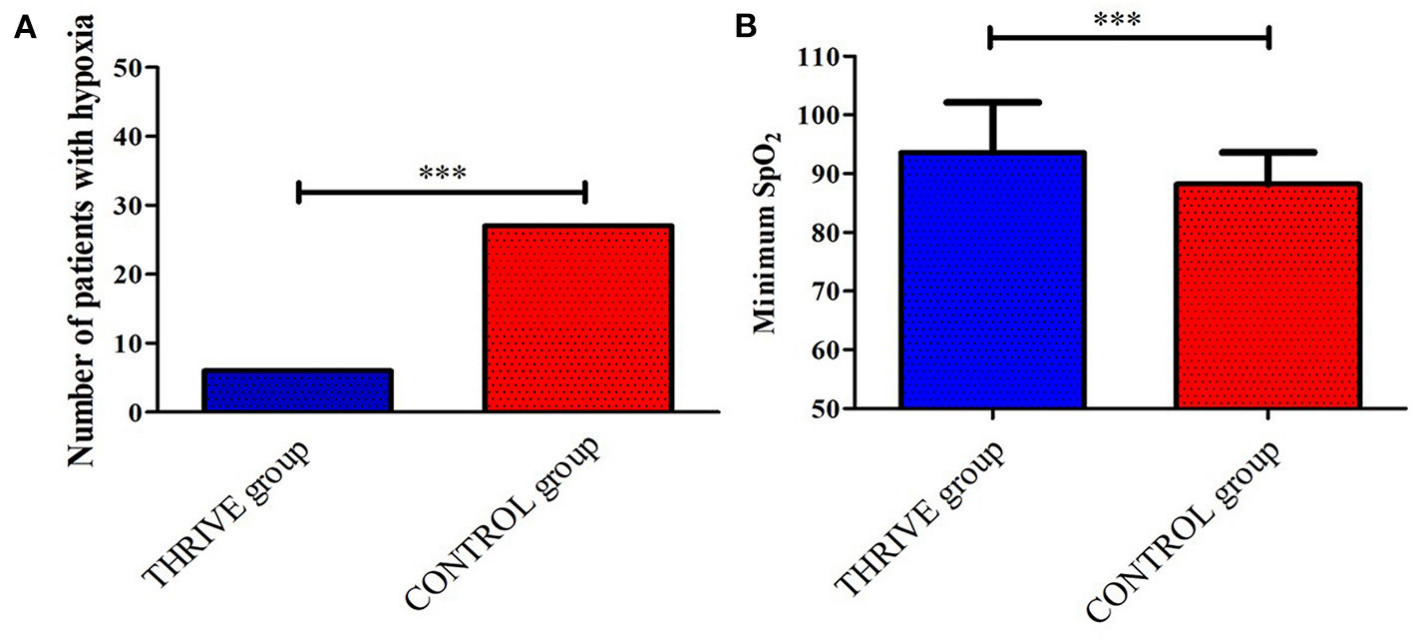
Between-group comparison of the number of patients who had desaturation **(A)** and the minimum SPO_2_ **(B)** during the stay in post-anesthesia care unit. ***P < 0.001.

As shown in Figure 2A, six (12%) patients in the THRIVE group and 27 (54%) patients in the CONTROL group experienced desaturation in the PACU. The THRIVE group showed a significantly lower incidence of desaturation during patients' stay in PACU than the CONTROL group (OR = 0.22 [95% CI, 0.10–0.49], *P* < 0.001). As shown in Figure 2B, patients in the THRIVE group showed a significantly higher minimum SpO_2_ than those in the CONTROL group (93.6 ± 8.5 vs. 88.2 ± 5.4, *P* < 0.001). In the THRIVE group, all patients with SpO2 <90% after tracheal extubation recovered to > 95% after jaw thrust. All patients in the CONTROL group with SpO_2_ <90% after tracheal extubation increased with their SpO_2_ > 95%, 19 patients after a deep breath and eight patients required supplemental oxygen *via* a nasal cannula.

**Figure 3:**
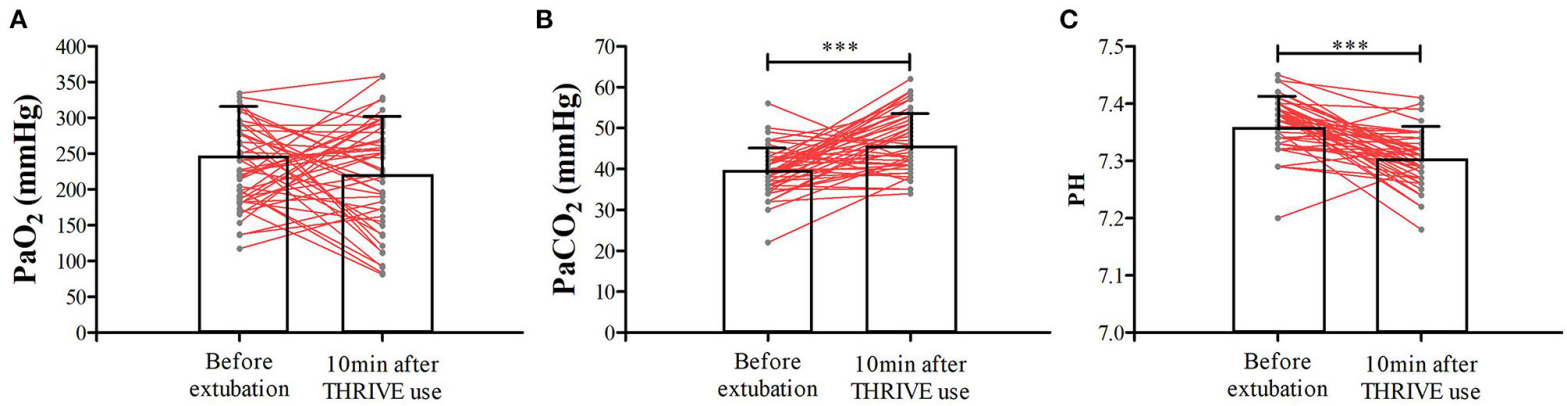
The PaO_2_ **(A)**, PaCO_2_ **(B)**, and PH **(C)** at the time of tracheal extubation and 10 min after THRIVE use for patients in the THRIVE group. ***P < 0.001 compared with before extubation.

In the THRIVE group, the mean PSI and TOF were 54.7 ± 12.7 and 56.5 ± 26.4%, respectively, when tracheal extubation was performed. THRIVE was applied for 22.9 ± 7.4 min. While in the CONTROL group, the mean PSI and TOF were 87.8 ± 6.1 and 94.0 ± 7.7%, respectively. Figure 3 shows data from the arterial blood gas analysis. In the THRIVE group, 10 min after the commencement of THRIVE, PaCO_2_ (46.9 ± 7.5 vs. 39.3 ± 5.6 mmHg, *P* < 0.001) increased and PH (7.36 ± 0.05 vs. 7.30 ± 0.05, *P* < 0.001) decreased compared to baseline before tracheal extubation; however, there was no significant between-point difference (10 min after THRIVE vs. before extubation) in PaO_2_ (246.6 ± 80.9 vs. 225.0 ± 74.9 mmHg, *P* = 0.138). At discharge from the PACU, PaCO_2_ and PaO_2_ were 40.6 ± 5.4 and 105.1 ± 38.9 mmHg, respectively. As shown in Table 2, EIT revealed no between-group differences for ROI ΔEELI and overall ΔEELI at discharge from the PACU compared with the time of admittance to the PACU (*P* > 0.05).

**Table 2:** The overall ΔEELI at the time of leaving PACU and the ΔEELI in different ROI area for different groups.

	**THRIVE group (*n* = 50)**	**CONTROL group (*n* = 50)**	***P***
ΔEELI1	−0.41(−0.66 ~−0.16)	−0.27(−0.72 ~−0.15)	0.463
ΔEELI2	−0.58(−1.09 ~−0.22)	−0.27(−0.91 ~ 0.25)	0.064
ΔEELI3	−0.12(−0.42 ~ 0.19)	−0.12(−0.41 ~ 0.20)	0.963
ΔEELI4	−0.16(−0.24 ~−0.08)	−0.24(−0.47 ~−0.14)	0.085
ΔEELIglo	−1.32(−2.24 ~−0.64)	−0.87(−2.48 ~−0.07)	0.264

*Values are presented as median (IQR [range])*.

**Figure 4:**
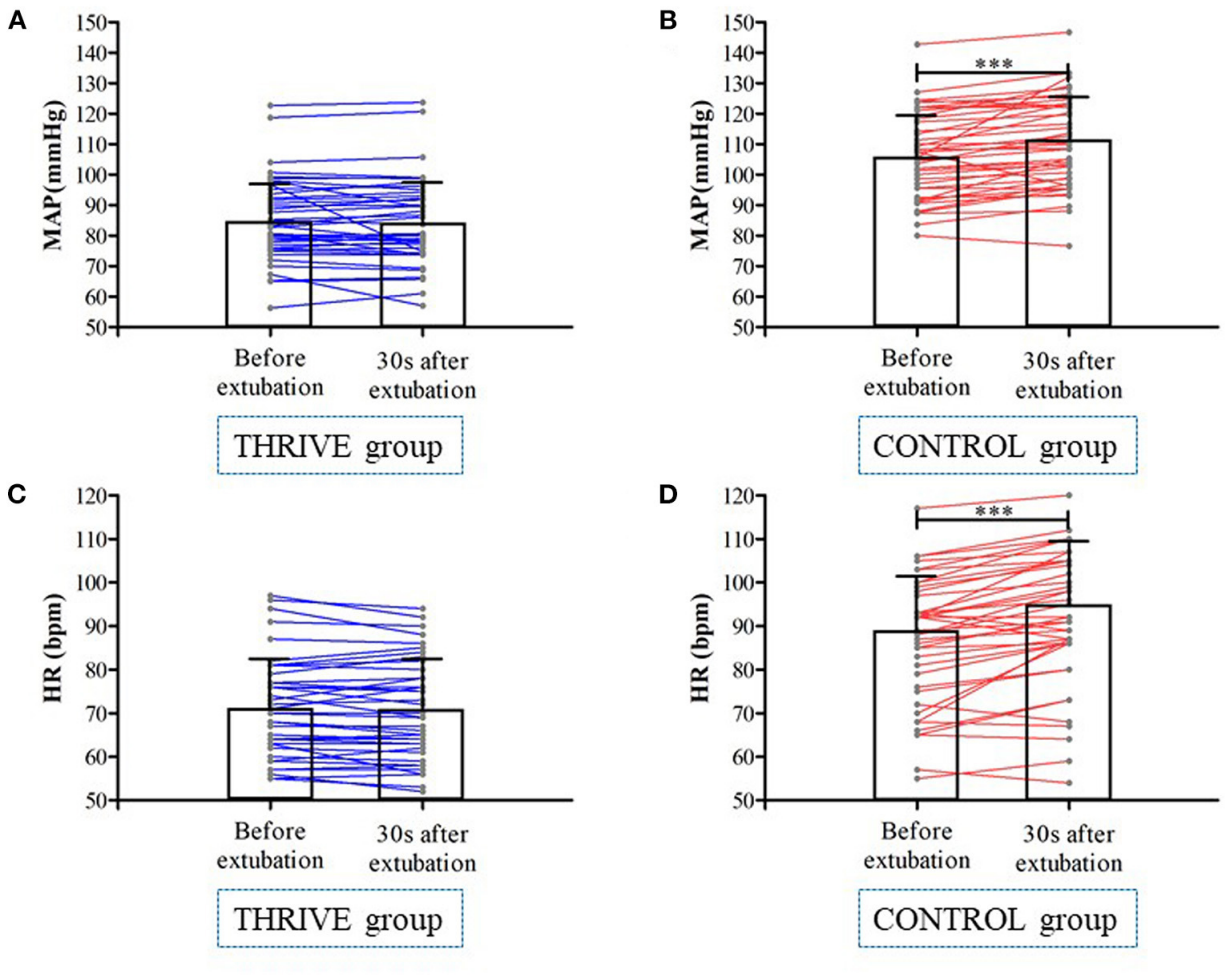
Mean arterial pressure **(A,B)** and heart rate **(C,D)** at before and 30 s after tracheal extubation in THRIVE and CONTROL group. ***P < 0.001.

In the THRIVE group, there was no significant change in MAP (84.3 ± 14.1 vs. 85.0 ± 13.5 bpm, *P* = 0.371) and heart rate (71.5 ± 11.7 vs. 70.9 ± 11.7 bpm, *P* = 0.190) at 30 s after extubation compared to before extubation (Figure 4). In contrast, in the CONTROL group, MAP (110.4 ± 14.7 vs. 106.3 ± 13.9 bpm, *P* < 0.001) and heart rate (92.6 ± 16.1 vs. 87.9 ± 15.5 bpm, *P* < 0.001) increased 30 s after extubation compared with pre-extubation (Figure 4). The THRIVE group had fewer patients with an at least 20% increase in MAP (4 vs. 26%, OR = 0.15 [95% CI, 0.04–0.65], *P* = 0.002) than the CONTROL group. There was no significant between-group difference in the number of patients showing an at least 20% increase in heart rate (32 vs. 36%, *P* = 0.672).

**Figure 5:**
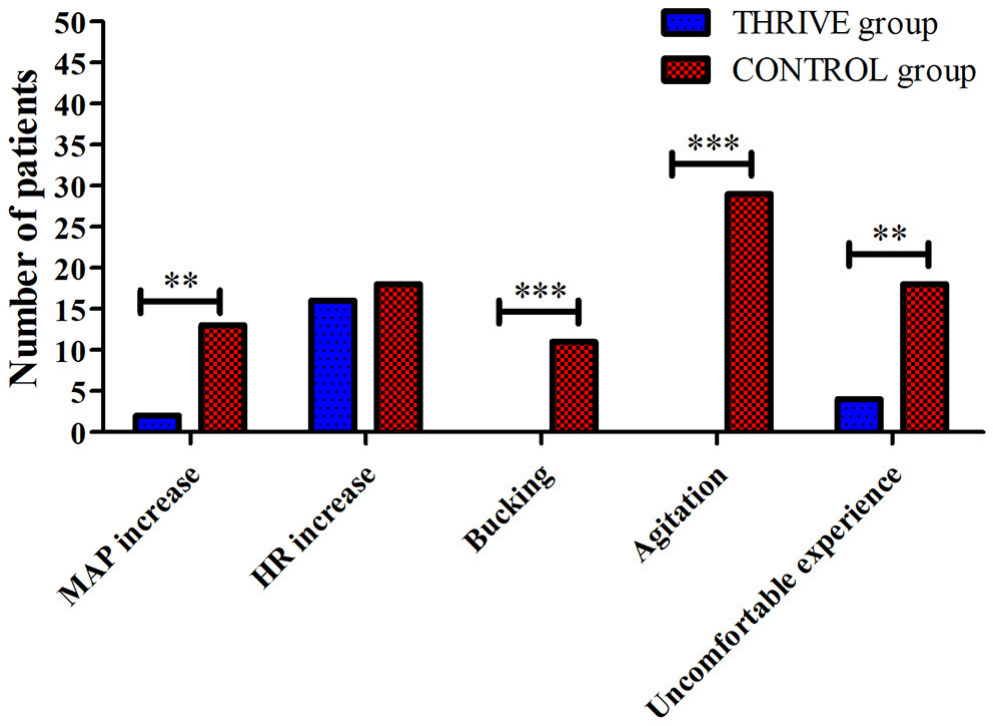
Between-group comparison of incidences of blood pressure and heart rate fluctuation, bucking and agitation, and subjective uncomfortable experience. HR, heart rate; MAP, mean arterial pressure, **P < 0.01, ***P < 0.001.

While admitted to the PACU, none of the patients in the THRIVE group showed coughing (0%) or agitation (0%); however, the incidence of cough and agitation in the CONTROL group was 22 and 58%, respectively (Figure 5). No other adverse events such as aspiration, bleeding, and reintubation was found in the patients of two groups. Furthermore, less patients in the THRIVE group compared to the CONTROL group reported an uncomfortable experience (8 vs. 36%, 0.22 [95% CI, 0.08–0.61], *P* < 0.001) during their stay in the PACU.

There were no between-group differences in postoperative data (Table 3), including the recovery quality score (119.7 ± 9.9 vs. 118.7 ± 10.8, *P* = 0.480), length of hospital stay (8.0 [6–13.8] vs. 7.0 [5–15.5] days, *P* = 0.908). However, the incidence of postoperative laryngalgia or hoarseness in the THRIVE group was half of that in the CONTROL group (14 vs. 28%, *P* = 0.085).

**Table 3:** The monitor parameters at the time of leaving PACU for patients in different groups.

	**THRIVE group (*n* = 50)**	**CONTROL group (*n* = 50)**	***P***
PACU duration (min)	56.2 ± 11.2	60.3 ± 16.7	0.146
Mean arterial pressure(mmHg)	92.9 ± 13.3	96.4 ± 12.6	0.186
Heart rate(bpm)	73.9 ± 12.1	77.2 ± 12.8	0.193
Cardiac output (L/min)	5.6 ± 1.6	6.2 ± 1.5	0.077
Systemic vascular resistance(dyn.s/cm^5^)	1368.2 ± 416.3	1227.8 ± 408.5	0.105
Stroke volume (ml)	76.8 ± 15.7	80.0 ± 15.9	0.329
Stroke volume variation (%)	7.6 ± 4.2	8.1 ± 3.8	0.542
rSO_2_L (%)	82.9 ± 6.6	83.1 ± 8.2	0.929
rSO_2_R (%)	83.8 ± 6.0	82.2 ± 3.9	0.360
Lac (mmol/L)	0.91 ± 0.39	0.99 ± 0.39	0.321
Train-of-four (%)	101.4 ± 10.3	104.1 ± 27.6	0.525
Patient state index	90.2 ± 6.7	90.8 ± 6.4	0.623
Respiratory rate (bpm)	14.3 ± 4.3	14.2 ± 4.3	0.944
PaO_2_ (mmHg)	105.1 ± 38.9	95.4 ± 22.3	0.130
PaCO_2_ (mmHg)	40.6 ± 5.4	39.8 ± 4.8	0.425
SaO_2_ (%)	98.2 ± 1.5	98.1 ± 1.2	0.866
FiO_2_(%)	22.8 ± 6.0	22.8 ± 3.9	1.000
OI	462.6 ± 125.2	421.9 ± 85.1	0.061
HCO3^−^(mmol/L)	22.0 ± 2.4	21.5 ± 2.1	0.235
SPO_2_ (%)	98.3 ± 2.1	97.8 ± 2.1	0.257

*SVR, Systemic vascular resistance; Values are presented as mean (SD)*.

## Discussion

This is the first randomized controlled study assessing the role of THRIVE to facilitate tracheal extubation under deep anesthesia in the PACU. Our findings showed that tracheal extubation employing THRIVE decreased the incidence of desaturation and hypertension, eliminated agitation and bucking, and allowed for a more comfortable experience in the PACU compared to awake extubation.

Commonly, tracheal extubation is performed when the patient is fully awake and able to sufficiently breath spontaneously (31, 32). However, extubation of conscious patients can be associated with agitation, bucking, hypertension, and tachycardia, all of which decrease the patients' quality of recovery (2–5). To prevent the unwanted side effects of awake tracheal extubation, extubation under deep anesthesia but with maintained spontaneous breathing was advocated (6–8). Previous clinical studies have shown that extubation under deep anesthesia decreases the stress response and improves the level of comfort (9–11). However, tracheal extubation in the unconscious patients increases the risk of hypoxaemia, aspiration or similar events and prolongs recovery time (12, 13).

THRIVE has been developed to provide continuous ventilatory support (33, 34) and has been shown to provide adequate oxygenation (24.1 ± 6.4 min) in patients undergoing non-intubated laryngomicrosurgery (24). In the current setting, patients' muscle relaxant is recovering after ending of general anesthesia, and we found that the average THIRVE time was 23 min in the study. Thus even if 56.5 ± 26.4% for TOF value is really low when extubation was performed in patients of THRIVE group, it is safe and feasible in the current setting. Our results reveal that 27 (54%) patients in the awake CONTROL group experienced desaturation. We thought the possible reason for the observed difference in incidence of desaturation between two groups was the transformational method. THRIVE provides patients with good oxygenation to recovery the patients from apnea to autonomous respiration. On the other hand, in CONTROL group direct transformation from mechanical ventilation to autonomous respiration was performed. In contrast, in the unconscious THRIVE group, only 6 (12%) patients showed a hypoxic event following extubation. The transitional respiratory support provided by THRIVE might be responsible for the decreased incidence of desaturation observed. Worth mentioning, in all six patients who presented with desaturation in the THRIVE group, SpO_2_ could be increased to 100% employing solely the jaw-thrust maneuver.

Although THRIVE allows for adequate oxygenation, carbon dioxide accumulation has been described (20, 35). In our study, arterial blood gas analysis revealed a significant increase in PaCO_2_ from 39.3 before to 46.9 mmHg 10 min after extubation with a maximum PaCO_2_ of 62 mmHg. Hua et al. reported that mean PaCO_2_ increased from ~40-72 mmHg after 10 min of THRIVE use (33). Recent studies reported that end-tidal carbon dioxide raised by 1.13-1.88 mmHg/min apnoea (19), with substantial variation (range: 0.98–2.63 mmHg/min) (18). In our study, mean PaCO_2_ increase was 0.7 mmHg/min, thus substantially slower than reported in existing literature. The slower rise in PaCO_2_ could be explained by a partial recovery of consciousness and spontaneous breathing in our patients, decreasing carbon dioxide accumulation. It must be emphasized that PaCO_2_ in the THRIVE group recovered to normal levels at discharge from the PACU. Furthermore, given the lack of difference in ΔEELI between admittance to and discharge from the PACU, THRIVE did not affect the pulmonary volume of patients in the THRIVE group. Our findings suggest that THRIVE, in a selected group of patients is safe; however, patient selection is crucial. Those patients prone to deteriorate from hypercapnia, e.g., patients with pulmonary hypertension or neurosurgical patients, should most likely not be treated with THRIVE for this indication.

The postoperative stress response is among the most expected adverse effects of awake extubation (36). Deep extubation has been developed to decrease the incidence of stress-related adverse effects, including agitation, bucking. Currently, deep extubation is mainly used in pediatric patients. A meta-analysis reported that the incidence of airway complications for pediatric patients remained at 40% when extubated under deep anesthesia (12). In adults, the incidence of airway complications, including bucking, for extubation under deep anesthesia was 13% (16). Our findings confirmed that bucking and agitation were effectively avoided in the THRIVE group, whereas in the CONTROL group bucking and agitation occurred in 22 and 58%, respectively. Moreover, the hypertension was significantly lower in the THRIVE group. Furthermore, the THRIVE group showed a significantly lower proportion of patients with an uncomfortable experience. Thus, we confirmed that tracheal extubation under deep anesthesia using THRIVE can effectively prevent stress responses and improve patients'comfort.

In addition, we did not find between-group difference in the quality of recover scores or length of hospital stay. However, the incidence of laryngalgia or hoarseness in the THRIVE group was half of that in the CONTROL group. Although the difference was not statically significant, the trend toward airway protection is in line with the lack of bucking and agitation in the THRIVE group. Therefore, there is a need for future large-scaled studies to examine the effects of tracheal extubation using THRIVE under deep anesthesia on laryngalgia or hoarseness.

This study has several limitations. First, we studied a highly selected patient population limiting generalisability of our findings to excluded groups of patients, for instance elderly patients. Second, given the nature of the intervention, it was not feasible to do a double-blinded study. However, outcomes were assessed by an independent investigator and data analysis was performed by a blinded statistician. Thirdly, PaCO_2_ was only monitored at several time points, and we could not assess the continuous changes in the values. Future studies should establish the combined use of non-invasive transcutaneous CO_2_ monitors. And adverse events such as aspiration/obstruction are often unpredictable and cannot be completely avoided (37), airway protection and related airway emergency plans must be considered during THRIVE application. Additionally, THRIVE should be used prudently for the purpose of extubation of a difficult airway; in these patients a B-plan for difficult extubation and eventual reintubation should be ready and set.

In conclusion, compared with traditional awake tracheal extubation in the PACU, extubation under deep anesthesia using THRIVE decreases the incidence of desaturation and provides a more comfortable experience to patients during their postoperative recovery, indicating that the current method is safe and effective for extubation during emergence from general anesthesia. Extubation under deep anesthesia using THRIVE might become an alternative strategy in those selected patient populations in demand for reducing the response to the extubation during emergence from general anesthesia.

## Data Availability Statement

The raw data supporting the conclusions of this article will be made available by the authors, without undue reservation.

## Ethics Statement

The studies involving human participants were reviewed and approved by Hospital Ethics Committee of the Second Affiliated Hospital, Chongqing Medical University. The patients/participants provided their written informed consent to participate in this study.

## Author Contributions

JQ, MX, and JC: data collection and writing up of the first draft of the paper. BC, YC, and XZ: patient recruitment and data collection. HL: data analysis. TZ: study design. GD: study design and revision of the draft of the paper. HH: study design and revision of the draft of the paper. All authors have read and approved the final manuscript.

## Funding

The study was supported by the Joint Medical Research Project of Chongqing Science and Technology Bureau and Health Commission (No. 2018ZDXM033) and National Key Research and Development Project (No. 2018YFC2001800).

## Conflict of Interest

The authors declare that the research was conducted in the absence of any commercial or financial relationships that could be construed as a potential conflict of interest.

## Publisher's Note

All claims expressed in this article are solely those of the authors and do not necessarily represent those of their affiliated organizations, or those of the publisher, the editors and the reviewers. Any product that may be evaluated in this article, or claim that may be made by its manufacturer, is not guaranteed or endorsed by the publisher.
